# Mitigation of the Toxic Effects of Periodontal Pathogens by Candidate Probiotics in Oral Keratinocytes, and in an Invertebrate Model

**DOI:** 10.3389/fmicb.2020.00999

**Published:** 2020-06-16

**Authors:** Raja Moman, Catherine A. O’Neill, Ruth G. Ledder, Tanaporn Cheesapcharoen, Andrew J. McBain

**Affiliations:** ^1^Department of Microbiology and Immunology, Faculty of Pharmacy, University of Tripoli, Tripoli, Libya; ^2^Division of Musculoskeletal and Dermatological Sciences, School of Biological Sciences, The University of Manchester, Manchester, United Kingdom; ^3^Division of Pharmacy and Optometry, School of Health Sciences, Faculty of Biology, Medicine and Health, The University of Manchester, Manchester, United Kingdom

**Keywords:** periodontal pathogens, *Galleria mellonella*, probiotics, infection model, keratinocytes

## Abstract

The larvae of the wax moth *Galleria mellonella* and human oral keratinocytes were used to investigate the protective activity of the candidate oral probiotics *Lactobacillus rhamnosus* GG (LHR), *Lactobacillus reuteri* (LR), and *Streptococcus salivarius* K-12 (SS) against the periodontal pathogens *Fusobacterium nucleatum* (FN), *Porphyromonas gingivalis* (PG), and *Aggregatibacter actinomycetemcomitans* (AA). Probiotics were delivered to the larvae (i) concomitantly with the pathogen in the same larval pro-leg; (ii) concomitantly with the pathogen in different pro-legs, and (iii) before inoculation with the pathogen in different pro-legs. Probiotics were delivered as viable cells, cell lysates or cell supernatants to the oral keratinocytes concomitantly with the pathogen. The periodontal pathogens killed at least 50% of larvae within 24 h although PG and FN were significantly more virulent than AA in the order *FN* > *PG* > *AA* and were also significantly lethal to mammalian cells. The candidate probiotics, however, were not lethal to the larvae or human oral keratinocytes at doses up to 10^7^ cells/larvae. Wax worm survival rates increased up to 60% for some probiotic/pathogen combinations compared with control larvae inoculated with pathogens only. SS was the most effective probiotic against FN challenge and LHR the least, in simultaneous administration and pre-treatment, SS and LR were generally the most protective against all pathogens (up to 60% survival). For *P. gingivalis*, LR > LHR > SS, and for *A. actinomycetemcomitans* SS > LHR and LR. Administering the candidate probiotics to human oral keratinocytes significantly decreased the toxic effects of the periodontal pathogens. In summary, the periodontal pathogens were variably lethal to *G. mellonella* and human oral keratinocytes and the candidate probiotics had measurable protective effects, which were greatest when administrated simultaneously with the periodontal pathogens, suggesting protective effects based on bacterial interaction, and providing a basis for mechanistic studies.

## Introduction

Periodontitis is a complex infectious disease associated with inflammation and the loss of periodontal attachment and bone support. It has several etiological and contributing factors such as the accumulation of biofilm and calculus (Wolf et al., 2005; Slots, 2017) and the presence of certain bacteria that have been identified as periodontal pathogens. There are several concepts of periodontal pathogenesis including the specific plaque hypothesis, which emphasizes the importance of specific bacteria (Nisha et al., 2017); the concept of keystone pathogens (Hajishengallis and Lamont, 2012) where a given bacterium exerts effects that are disproportionate to its abundance, and the polymicrobial synergy and dysbiosis model (Lamont and Hajishengallis, 2015; Nisha et al., 2017) all of which are significantly related to oral bacteria. Gram-negative anaerobic bacteria, in particular, have been implicated in the etiology of periodontitis. *Aggregatibacter actinomycetemcomitans, Fusobacterium nucleatum*, and *Porphyromonas gingivalis*, as well as other bacterial species including *Tannerella forsythia* and *Treponema denticola* (Socransky et al., 1998) are considered to be the important contributors to periodontitis in humans (Slots et al., 1986; Dzink et al., 1988; Duncan et al., 1993; Sandros et al., 1994).

*A. actinomycetemcomitans* is reported to damage host tissue via the production of a leukotoxin (Johansson, 2011), and a cytolethal distending toxin (DiRienzo, 2014). *F. nucleatum* directly influences host responses and can also increase the infectivity of other pathogens via the induction of expression of the antimicrobial peptide β-defensin and pro-inflammatory cytokines in the oral epithelium (Krisanaprakornkit et al., 2000; Bhattacharyya et al., 2016; Ahn et al., 2017). *P. gingivalis* expresses two types of gingipains (Imamura, 2003), which are reportedly implicated in the progression of periodontal disease and have been strongly associated with the induction of inflammation and destruction of the host periodontium (Miyachi et al., 2007). *Porphyromonas gingivalis* has been linked to the perturbation of periodontal microbial homeostasis. Hence, this bacterium has been proposed as a keystone periodontal pathogen (Hajishengallis and Lamont, 2012).

The potential of putatively beneficial bacteria (probiotics) to prevent or treat periodontitis and other oral diseases has been investigated (Vivekananda et al., 2010; Ince et al., 2015; Bohora and Kokate, 2017; Brignardello-Petersen, 2017; Kobayashi et al., 2017). Previous investigations have demonstrated the ability of certain species of *Lactobacillus* to inhibit the growth of *P. gingivalis* and *A. actinomycetemcomitans* (Sookkhee et al., 2001; Kõll-Klais et al., 2005). Krasse et al. (2006) reported decreased gum bleeding and reduced gingivitis following the administration of a *Lactobacillus reuteri-*based candidate probiotic suggesting mechanisms including the production of bacteriocins such as reuterin, competition with oral pathogens, and anti-inflammatory activity (Krasse et al., 2006). Another study proposed that *Lactobacillus rhamnosus* GG could suppress bone loss in a mouse model of induced periodontitis (Gatej et al., 2018). The administration of *S. salivarius* K12 has been associated with reduced alveolar bone loss and resorption in a murine periodontitis model (Zhu et al., 2019).

The use of alternative animal models for studying pathogenic microorganisms has increased in recent years (Swanson and Hammer, 2000; Casadevall, 2005; Chamilos et al., 2011) for several reasons, including throughput, cost, and ethics (Ball et al., 1995). In addition to the fact that microbial virulence mechanisms may be common between different hosts (Rahme et al., 1995), larvae are simple to work with, inherently replicable, and have a short life cycle in comparison to higher animals (Tsai et al., 2016). The presence of an innate immune system in invertebrates (Boman and Hultmark, 1987; Tsai et al., 2016) is an additional advantage over the use of alternatives such as cell culture.

*Galleria mellonella*, the caterpillar (larva) of the greater wax moth *(Lepidoptera: pyralidae*) is widely used as a non-mammalian animal model system to study host-pathogen interactions using a variety of microorganisms including bacteria (Morton et al., 1983; Miyata et al., 2003; Fedhila et al., 2006; Aperis et al., 2007; Seed and Dennis, 2008) and fungi (Mylonakis et al., 2005). Use of the *G. mellonella* model to study the protective effects of candidate probiotics has received some research attention where for example, it has been applied in the evaluation of candidate probiotics to impair *Pseudomonas aeruginosa* biofilm formation (Berrios et al., 2018), to reduce virulence in *Candida albicans* (Vilela et al., 2015; de Oliveira et al., 2017; Rossoni et al., 2017, 2018), and to protect against gastrointestinal pathogens such as *Listeria monocytogenes* and *Escherichia coli* (Scalfaro et al., 2017). Significant correlations between observed virulence in *G. mellonella* and mammalian models have been previously reported (Jander et al., 2000; Mylonakis et al., 2005), and *G. mellonella* host immune defense mechanisms have been proposed to broadly resemble those of humans (Nathan, 2014; Kohler, 2015). Host responses of the larvae can be assessed through both the cellular response mediated by phagocytic cells and the humoral immune response pathway mediated by antimicrobial peptides (AMPs) (Kavanagh and Reeves, 2004; Scalfaro et al., 2017).

The current study aimed to study the interactions between candidate probiotics *(L. rhamnosus* GG, *L. reuteri*, and *S. salivarius* K12) and periodontopathogens *(A. actinomycetemcomitans, F. nucleatum*, and *P. gingivalis)* in oral keratinocytes and a lower animal infection model (*G. mellonella*).

## Materials and Methods

### Bacterial Strains and Culture Preparations

Candidate probiotics comprised *Lactobacillus rhamnosus* Goldin and Gorbach (GG) (ATCC 53103), *Lactobacillus reuteri* ATCC 55730, and *Streptococcus salivarius* K-12. The periodontal pathogens were *Fusobacterium nucleatum* ATCC 10953, *Porphyromonas gingivalis* ATCC 33277, and *Aggregatibacter actinomycetemcomitans* ATCC 33384. *Aggregatibacter actinomycetemcomitans* was grown in Tryptic Soya Agar and broth supplemented with 0.6% yeast extract (TSA and TSB) and incubated in a 5% CO_2_ atmosphere. All other bacteria were grown using Wilkins-Chalgren broth or agar (Oxoid, Basingstoke, United Kingdom) at 37°C and incubated in an anaerobic cabinet (atmosphere, 10:10:80, H_2_, CO_2_, N_2_). All bacteria were incubated at 37°C. For both *in vitro* and *in vivo* experiments, 10 ml volumes of each bacterium were prepared by growing cells overnight either anaerobically or in a 5% CO_2_ environment. A 100-fold dilution of overnight culture was made in sterile broth, which was incubated until the desired growth phase was reached according to constructed growth curves. Cells were harvested by centrifugation at 3220 × *g* for 15 min and the pelleted cells were washed and re-suspended in sterile phosphate buffer saline (PBS) (0.01 M PBS; NaCl 8 g/L, KCL 0.2 g/L, Na_2_HPO_4_ 1.42 g/L, and KH_2_PO_4_ 0.24 g/L, pH 7.4). These steps were repeated twice, and the cell suspension adjusted to a final optical density at 600 nm of 0.1. Each larva received aliquots of 5 μl of this bacterial suspension injected directly to the hemocoel. For experiments using lysates and cell-free extracts, 10 ml of 10^8^ Colony Forming Units (CFU)/ml of the appropriate strain was centrifuged. The supernatant was reserved to use as a cell-free extract. The pellet was washed, concentrated in 1 ml of Wilkins Chalgren broth, and lysed using a bead beater (FastPrep FP120; Thermo Electron Corporation). Samples were filter sterilized to remove any whole bacteria remaining.

### *Galleria* Infection Model

*G. mellonella* were obtained from Live Foods Direct, Sheffield, United Kingdom. The larvae were in the last instar stage (shedding of the exoskeleton), and were selected based on their weight (275–300 mg), the presence of a fresh cream color, and no gray markings. All larvae were used within 3 days from shipment.

### Well-Diffusion Test

The test organisms were grown to the stationary phase (10^8^ CFU/ml). Cultures of pathogenic bacteria were diluted 1:100 in Wilkins Chalgren agar. After careful mixing, 20 ml agar plates were poured and left to set. Once set, cup cuts were aseptically made within the agar (8 mm wells) and filled with 100 μl of the probiotic cell culture (∼1.5 × 10^8^ CFU/ml) of each tested probiotic organism. The plates were incubated at 37°C anaerobically for 48 h and the diameter of the zone of inhibition produced measured using calipers.

### Human Oral Keratinocyte Cell Culture

Human oral keratinocytes (HOKs, Sciencell Research Laboratories, United States) were used to assess the effect of probiotics on their viability. HOKs were maintained in oral keratinocyte medium (OKM, Sciencell Research Laboratories, United States) supplemented with oral keratinocyte growth supplement (OKGS) and 100 U/ml of both penicillin and streptomycin (OKM, Sciencell Research Laboratories, United States). The medium was substituted twice weekly and cells were incubated in a humid atmosphere with 5% CO_2_ at 37°C. Cells were cultured in T-25 or T-75 vented culture flasks and 24 well plates (Corning, Sigma, United States). The cells were plated, at a density of 10 × 10^4^ cells per cm^2^, in 1 ml of the appropriate medium either in 12 or 24 well plates according to the experiment and used after 24 h incubation at 37°C at ∼90–100% confluence. Cells were exposed to 10^8^ CFU/ml of each probiotic cell suspension for 24 h. Viability was determined using the trypan blue exclusion assay (Prince et al., 2012). Uninfected cells were included as a control. Probiotic lysates and cell-free extracts (100 μl) were added simultaneously with the periodontopathogen to the human oral keratinocytes.

### *Galleria mellonella* Pathogenicity and Protection Assays

A modified version of the assay described by Ramarao et al. (2012) was performed. Larvae of *G. mellonella* were incubated for 30 min at room temperature before injection. Overnight cultures of each microorganism were centrifuged (3220 × *g*, 15 min) and suspended in PBS. This was repeated twice. Cultures were adjusted to an OD_600 nm_ of 0.1. For intrahemocoelic injection, bacterial suspensions were prepared with final concentrations in the range of 10^4^ CFU/ml to 10^8^ CFU/ml. Volumes of 5 μl of each strain, cell-free extract or lysate were delivered directly to the hemocoel through an injection in the rear left pro-leg using a 26-gauge needle Hamilton microsyringe (Sigma, United Kingdom). Sterile PBS (5 μl) was injected into the “trauma” control group and additionally, a “no treatment” control group was added. The right pro-leg was used as the injection site. Different sites were used for pathogenic and probiotic strains to reduce the risk of injection site infection. Infected larvae were incubated in a petri dish in groups of 10 at 37°C in the dark for the duration of the experiment (5–7 days).

### Determination of Larval Mortality

Larval mortality was determined daily over a week. Larvae that had turned black and that were not moving in response to a gentle shaking of the dish or touching with a pipette tip were considered dead. Dead larvae were removed from the petri dish and the death was recorded. The experimental endpoint was designated by either the death of all the larvae in the tested groups or the conversion of larvae into pupae. Pupae were identified via a color change to white (Jorjao et al., 2018). Five Petri dishes containing 10 larvae each were assigned to each experiment and control groups (50 larvae total for each sample). Dead *G. mellonella* were placed into sterile Universal bottles and homogenized in 10 ml of sterile PBS. This suspension was then serially diluted, and spot plated onto Wilkins Chalgren agar to calculate bacterial load per individual larva. The experiments were terminated once two of the control individuals had died or pupated.

### Statistical Analyses

*Galleria mellonella* data were plotted as survival curves using the Kaplan–Meier estimator in Microsoft Excel 2010. The survival values were considered significantly different if the *p*-value was < 0.05. For cell culture work paired student’s *T*-tests done using Microsoft Excel 2010. Results were considered significant if *p* ≤ 0.05.

## Results

### *In vitro* Antibacterial Activity of Selected Probiotics

*In vitro* testing of antibacterial activity using an agar well-diffusion assay indicated that all three tested pathogens were inhibited by the three investigated probiotics to varying degrees. *A. actinomycetemcomitans* showed the greatest sensitivity to all probiotics with significantly larger zones of inhibition produced than for other pathogens. *Streptococcus salivarius* K-12 was the most effective against *A. actinomycetemcomitans* (Table 1). For both *F. nucleatum* and *P. gingivalis* the effect of all probiotics was comparable with *P. gingivalis* exhibiting slightly more sensitivity (Table 1).

**TABLE 1:** Inhibition of periodontal pathogens by suspensions of candidate probiotics.

**Periodontal pathogen**	**Candidate probiotic**		
	***L. rhamnosus***	***L. reuteri***	***S. salivarius* K-12**
*P. gingivalis*	19 ± 1	15 ± 2	20 ± 2
*F. nucleatum*	11 ± 2	15 ± 1	20 ± 0
*A. actinomycetemcomitans*	33 ± 2	24 ± 0	41 ± 2

**Determined using agar well-diffusion assays. Values are zones of inhibition (mm) and represent mean ± SEM from three separate experiments.**

### The Susceptibility of Human Oral Keratinocytes and *G. mellonella* to Periodontal Pathogens

**FIGURE 1:**
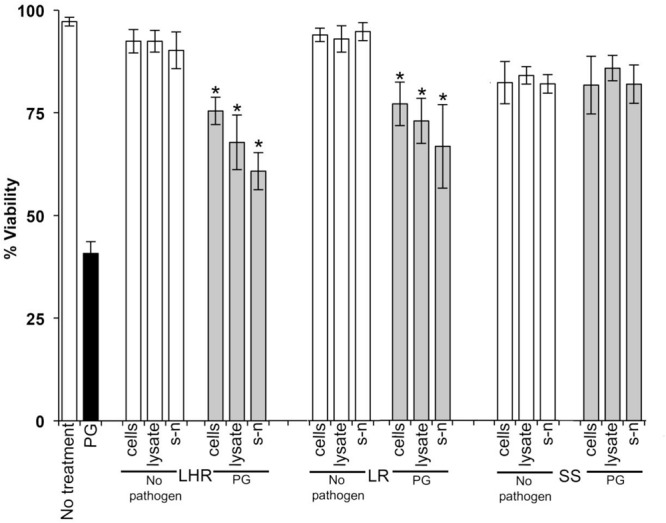
The protective effects of candidate probiotics for human oral keratinocytes (HOKs) against *P. gingivalis.* All probiotic cells, cell lysates, and culture supernatants protected HOKs against *P. gingivalis*. HOKs were inoculated with either live *L. rhamnosus* GG (LHR), *L. reuteri* (LR), or *S. salivarius* K*-*12 (SS) cells, lysates and supernatants (s-n) (white bars) or simultaneously in combination with *P. gingivalis* (PG) (gray bars). All experiments were performed for a minimum of three biological replicates, with three technical replicates each time. All data are shown as mean values plus/minus standard deviations. Results expressed as the mean ± SEM, *p < 0.05.

**FIGURE 2:**
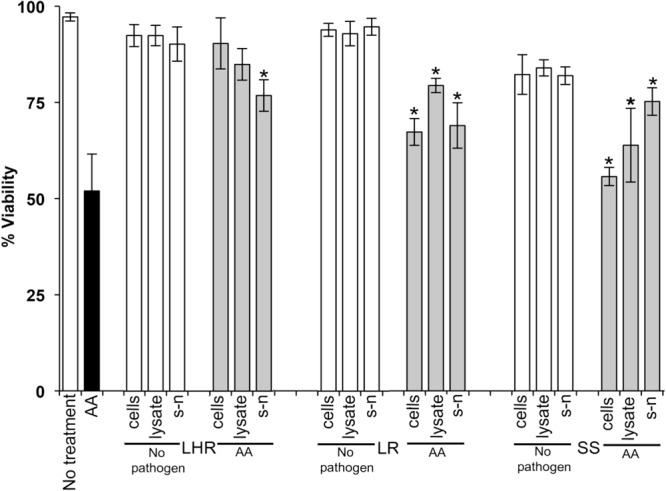
The protective effects of candidate probiotics for human oral keratinocytes against *A. actinomycetemcomitans.* All probiotic cells, cell lysates, and culture supernatants protect HOKs against *A. actinomycetemcomitans*. HOKs were inoculated with either live *L. rhamnosus* GG (LHR), *L. reuteri* (LR), or *S. salivarius* K*-*12 (SS) cells, lysates and supernatants (s-n) (white bars) or simultaneously in combination with *A. actinomycetemcomitans* (AA) (gray bars). All experiments were performed for a minimum of three biological replicates, with three technical replicates each time. All data are shown as mean values plus/minus standard deviations. Results expressed as the mean ± SEM, *p < 0.05.

**FIGURE 3:**
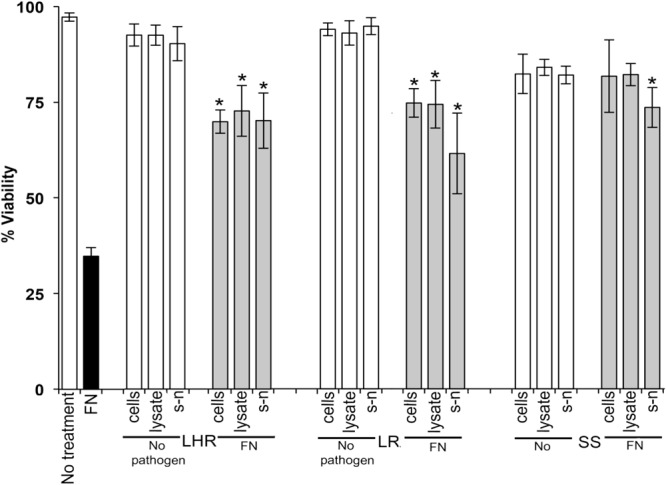
The protective effects of candidate probiotics for human oral keratinocytes against *F. nucleatum.* All probiotic cells, cell lysates, and culture supernatants protect HOKs against *F. nucleatum*. HOKs were inoculated with either live *L. rhamnosus* GG (LHR), *L. reuteri* (LR), or *S. salivarius* K−12 (SS) lysates and supernatants (s-n) (white bars) or simultaneously in combination with *F. nucleatum* (FN) (gray bars). All experiments were performed for a minimum of three biological replicates, with three technical replicates each time. All data are shown as mean values plus/minus standard deviations. Results expressed as the mean ± SEM, *p < 0.05.

Data in Figures 1–3 show when control HOKs were incubated for 24 h, ∼97% ± 0.2 of the cells remained viable; whereas the percentage of cells that remained viable following 24 h inoculation with the periodontopathogens was significantly (*p* > 0.01) lower. *P. gingivalis* decreased the viability of treated cell monolayers to 40% (Figure 1), *A*. *actinomycetemcomitans* decreased the viability of cell monolayers to 51% (Figure 2), and *F. nucleatum* decreased the viability of treated cell monolayers to 34% (Figure 3).

**FIGURE 4:**
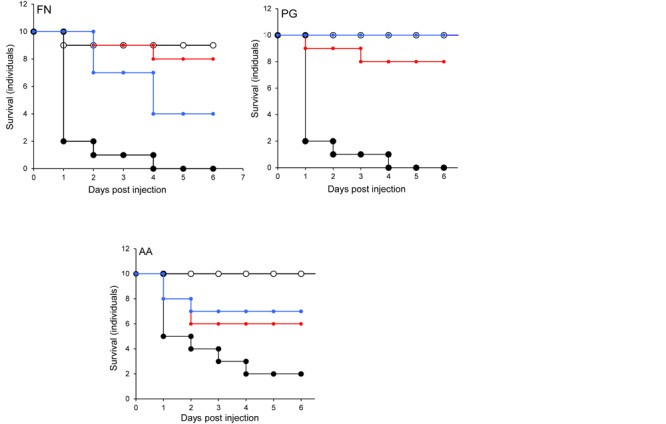
The lethal effect of periodontal pathogens in an invertebrate model. Kaplan-Meier plots of survival of *G. mellonella* larvae after challenge with periodontal pathogens. *F. nucleatum* (FN), *Porphyromonas gingivalis* (PG), or *A. actinomycetemcomitans* (AA). Viable bacterial suspension (black symbols), cell-free culture supernatant (blue symbols), bacterial lysate (red symbols), and PBS (white symbols). All experiments were done twice on 2 consecutive weeks, with different batches of larvae and with three replica plates where each plate contains 10 larvae. All three periodontal pathogens caused at least 50% larvae morality at the endpoint of the experiment. The mortality effects of *F. nucleatum* and *P. gingivalis* were significantly greater than *A. actinomycetemcomitans* (*p* < 0.05) in the order *F. nucleatum* > *P. gingivalis* > *A*. *actinomycetemcomitans*. Bacterial lysates and cell-free culture supernatants contributed to the reduction of larvae viability, but the lethal effects were lower than for viable bacteria.

In the *G. mellonella* model (Figure 4), no mortality was observed in either control (non-treated control or PBS control). All three pathogens caused the death of at least 50% of larvae by the experimental endpoint. However, *P. gingivalis* and *F. nucleatum* caused significantly higher mortality (*p* < 0.05) than *A*. *actinomycetemcomitans* in the order *F. nucleatum* > *P. gingivalis* > *A*. *actinomycetemcomitans* (Figure 4). Bacterial lysates and broth culture filtrates of pathogens reduced the viability of larvae, but the mortality rate was less than with live bacteria. Lysates were more lethal than cell-free extracts. These data show that HOKs and *G. mellonella* are susceptible to infection with selected periodontal pathogens. By contrast, none of the candidate probiotics (*L. rhamnosus* GG, *L. reuteri*, and *S. salivarius* K-12) induced significant mortality in HOKs or *G. mellonella* (Figures 1–4).

### Species-Dependent Protective Effects of Candidate Probiotics in *G. mellonella* and Human Oral Cell Lines Challenged With Periodontal Pathogens

**FIGURE 5:**
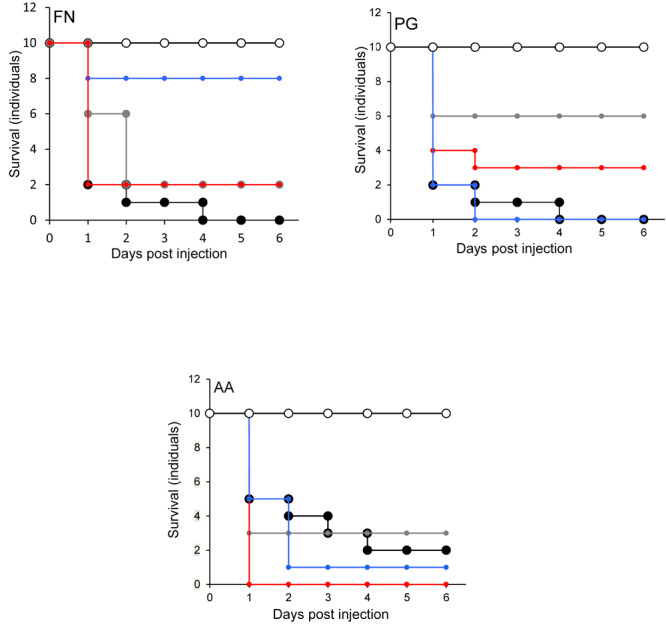
Protection of larvae conferred by candidate probiotics following simultaneous administration of periodontal pathogens into the same larval proleg. Pathogen only (black symbols) and PBS (white symbols). Pathogen administered simultaneously with *L. rhamnosus* (gray symbols), *L. reuteri* (red symbols), *S. salivarius* (blue symbols). All experiments were done twice on 2 consecutive weeks, with different batches of larvae and with three replica plates where each plate contains 10 larvae. *S. salivarius* K-12 had a protective effect (*p* ≤ 0.01) when administered simultaneously with *F. nucleatum* but not for *A. actinomycetemcomitans* or *P. gingivalis*. *L. reuteri* conferred protection against *P. gingivalis*, limited protection against *F. nucleatum*, and was not protective against *A. actimomycetemcomitans*. *L. rhamnosus* GG had some protective effect on larvae when was injected with *F. nucleatum* and *P. gingivalis* but reduced the larval viability when injected combined with *A. actinomycetemcomitans.*

When *S. salivarius* K-12 was injected with either *F. nucleatum* or *A. actinomycetemcomitans* there was a higher larval viability 24 h post-injection (*p* ≤ 0.01) than when *G. mellonella* was injected with the pathogens alone (Figure 5). However, *S. salivarius* K-12 did not protect larvae from the effects of *P. gingivalis* (Figure 5). *L. reuteri* afforded some protection against *P. gingivalis* but had a limited effect against *F. nucleatum* and *A. actinomycetemcomitans*. *L. rhamnosus* GG had some protective effect on *G. mellonella* viability when injected in a mixture with *F. nucleatum* and *P. gingivalis* but increased the mortality of larvae when injected in a mixture with *A. actinomycetemcomitans* (Figure 5). Probiotic protection in *G. mellonella* according to bacterial species was in the following order: for *F. nucleatum* SS > LR > LHR, for *P. gingivalis* LR > LHR > SS, and for *A. actinomycetemcomitans*, SS > LHR = LR. Administering probiotic strains, their supernatants and lysates simultaneously with *F. nucleatum* (Figure 3) or *P. gingivalis* (Figure 1) to human oral keratinocytes significantly (*p* < 0.05) increased the viability compared to when infected with the pathogen alone. Protection against *A. actinomycetemcomitans* inoculation in HOKs (Figure 2) was variable and was only statistically significant (*p* < 0.05) when lysates or supernatants were administered (*p* < 0.05).

### Effect of Injecting of Pathogen and Probiotic Simultaneously in Different Pro-legs on *G. mellonella* Mortality

**FIGURE 6:**
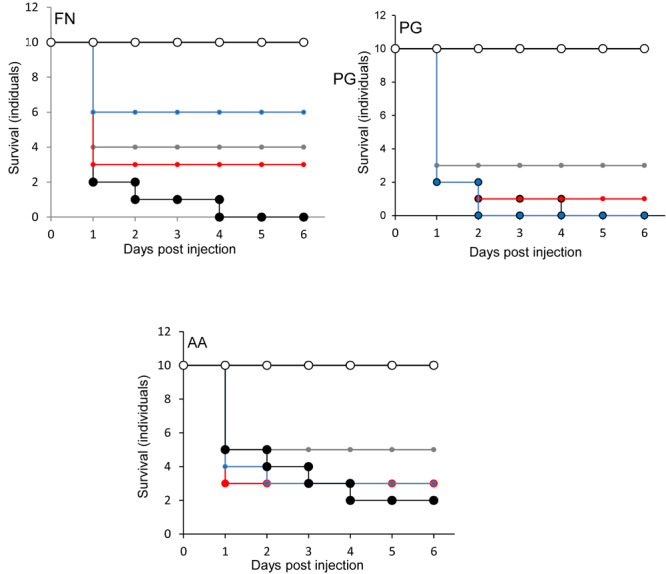
Protection of larvae conferred by candidate probiotics following simultaneous administration of periodontal pathogens into the different larval prolegs. Pathogen only (black symbols) and PBS (white symbols). Pathogen administered simultaneously with *L. rhamnosus* (gray symbols), *L. reuteri* (red symbols), or *S. salivarius* (blue symbols). All experiments were done twice on 2 consecutive weeks, with different batches of larvae and with three replica plates where each plate contains 10 larvae. The greatest protection against *F. nucleatum* was conferred by *S. salivarius* K-12. For *P. gingivalis* and *A. actinomycetemcomitans*. *L. rhamnosus* GG was the most protective bacterium.

The effects of injecting pathogens and probiotics into separate prolegs were evaluated. This was to exclude any effects due to an inhibitory interaction between microorganisms. The viability of larvae injected with *F. nucleatum* was increased when injected simultaneously with all probiotics. The most effective was *S. salivarius* K-12 (Figure 6). For *P. gingivalis, L. rhamnosus* GG reduced the lethal effect of *G. mellonella* (Figure 6), while with the other two probiotics the viability was lower than the larvae injected with *P. gingivalis* alone. *L. reuteri* decreased conferred protection against *A. actinomycetemcomitans*, as did *L. rhamnosus* GG and *S. salivarius* K-12 to a lesser extent (Figure 6). Probiotic protection according to bacterial species occurred in the following order: SS > LR > LHR for *F. nucleatum*, LR > LHR > SS for *P. gingivalis*, and LR > LHR = SS for *A. actinomycetemcomitans.*

### Effect of Probiotic Pre-treatment on Survival of *G. mellonella* Inoculated With Periodontal Pathogens

**FIGURE 7:**
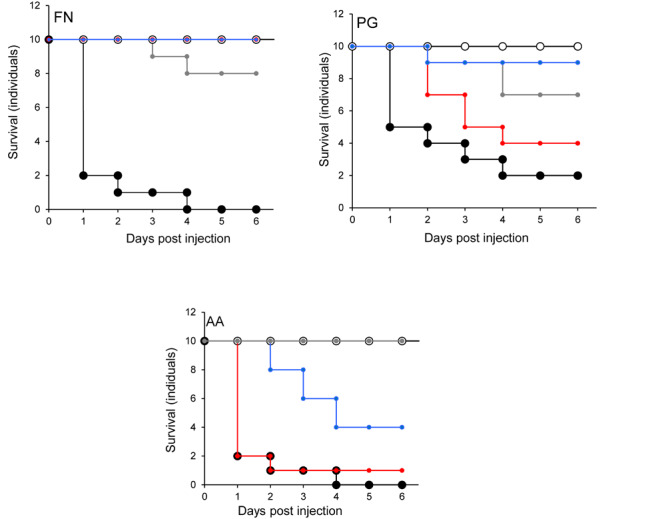
Protection of larvae conferred by prior inoculation with candidate probiotics and administration of periodontal pathogens 24 h later, into the different larval prolegs. Pathogen only (black symbols) and PBS (white symbols). *L. rhamnosus* (gray symbols), *L. reuteri* (red symbols), or *S. salivarius* (blue symbols). All experiments were done twice on 2 consecutive weeks, with different batches of larvae and with three replica plates where each plate contains 10 larvae. Prior-injection of larvae with *L. rhamnosus* GG or *S. salivarius* K-12 was highly protective against *F. nucleatum*. *L. reuteri* had the most effective protection against the effects of *P. gingivalis*, while *S. salivarius* afforded the greatest protective effect against *A. actinomycetamcomitans*.

None of the larvae were killed when *F. nucleatum* was injected into larvae that were pre-injected with *L. rhamnosus* GG or *S. salivarius* K-12 (Figure 7). Furthermore, there was a highly significant decrease (*p* < 0.001) in the mortality of larvae pre-treated with *L. reuteri* (Figure 7). The effects of injecting *P. gingivalis* on larvae that were pre-treated for 24 h with probiotics was total protection for larvae pre-treated with *L. reuteri* whereas the least protection was conferred by *L. rhamnosus* GG (Figure 7). For *A. actinomycetemcomitans* the best protection was conferred by *S. salivarius* K-12 followed by *L. reuteri* and *L. rhamnosus* GG was the least effective (Figure 7) in terms of the increase of viability of larvae compared to larvae treated by pathogen only (*p* < 0.01). Probiotic protection according to bacterial species was in the following order: LHR > SS > LR for *F. nucleatum*, LR > SS > LHR for *P. gingivalis*, and SS > LR > LHR for *A. actinomycetemcomitans.*

## Discussion

We have utilized a waxworm larval pathogenicity model, and human oral keratinocytes to study interactions between candidate probiotics and periodontal pathogens. *A. actinomycetemcomitans, F. nucleatum*, and *P. gingivalis* were variably lethal to the larvae and were significantly lethal in oral keratinocytes. The candidate probiotics were not significantly pathogenic in all infection models investigated. Inoculation of larvae with the candidate probiotica before pathogen challenge gave measurable but partial protection against the periodontopathogens. Protection in mammalian culture was conferred by bacterial cells, their lysates, and cell-free extracts. There are two main mechanisms by which this protection could be conferred. The first involves direct inhibition of the periodontal pathogens by the candidate probiotics. In this respect, we observed an antagonistic effect of the probiotics against the periodontopathogens in *in vitro* tests, where *A. actinomycetemcomitans* was inhibited to the greatest extent. Previous studies have demonstrated the susceptibility of this bacterium to probiosis both *in vitro* (Jaffar et al., 2016, 2018) and *in vivo* (Morales et al., 2018). However, protection of the waxworm could also be conferred by host-dependent mechanisms. The lethality of the pathogens in the *G. mellonella* model followed the order *F. nucleatum* > *P. gingivalis* > *A*. *actinomycetemcomitans. F. nucleatum*, and *P. gingivalis* demonstrated significantly greater pathogenicity than *A. actinomycetemcomitans.* This finding is broadly in agreement with the Socranksy complexes (Socransky et al., 1998) where *P. gingivalis* and *F. nucleatum* form part of the red and orange complexes respectively, which have been strongly associated with periodontal disease. The toxic effects of spent culture fluid and lysates were also investigated. Both were shown to affect the viability of the larvae but to a lesser extent than with inoculation with live bacteria. This could involve extra-cellular virulence factors in addition to those that require a viable cell to be present. For example, the production of sialidase in *P. gingivalis* (Frey et al., 2019), the induction of inflammation by *F. nucleatum* (Han, 2015), and the production of toxins in *A. actinomycetemcomitans* (Belibasakis et al., 2019).

All of the candidate probiotic strains tested conferred a degree of protection to infection. However, the extent of this protection was dependent on the species of probiotic and the pathogen under test. This is in agreement with other studies demonstrating strain and species-dependent effects of probiotics when targeted toward periodontal pathogens (Teughels et al., 2013; Montero et al., 2017). The direct protection observed could be due to competition for adhesion, acid production, production of bacteriocins, and biosurfactants (Spurbeck and Arvidson, 2010; Kohler et al., 2012; Orsi et al., 2014; Sabia et al., 2014).

The effects of simultaneously administering the probiotic strain and the pathogen to *G. mellonella* but in a different pro-legs were investigated to differentiate between direct competitive effects (as seen in Table 1) and immunomodulation. Data in Figure 6 suggest that there was a protective effect comparable to that observed when the probiotics were administered to the same site. This supports the hypothesis that the probiotics protect the larvae from infection *via* a mechanism distinct from direct competition, such as immunomodulation (Toshimitsu et al., 2017). Data in Figure 7 show the effects of administering the probiotics 24 h before pathogen inoculation. Competitive inhibition of pathogens by probiotic strains has previously been indicated as important in terms of observing a significant and relevant probiotic effect (Zhu et al., 2010; Munoz-Quezada et al., 2013). Figure 7 shows that for *F. nucleatum* injected 24 h after *Lactobacillus rhamnosus* administration, *F. nucleatum* injected after *S. salivarius* K12 and *P. gingivalis* injected after *L. reuteri* administration larval mortality was comparable to that observed in the PBS control group. This could be due to immunomodulation in *G. mellonella* that subsequently inhibited infection with the periodontopathogens. This observation is in agreement with a previous study that demonstrated that the experimental co-infection of *G. mellonella* with *L. acidophilus* and *C. albicans* reduced the number of yeast cells in the larval hemolymph and increased the survival of larvae (Ribeiro et al., 2017).

The *G. mellonella* larvae represent a cost-effective simple *in vivo* model as a preliminary investigative tool for screening the potential protective probiotic effects against periodontal and potentially other pathogens. Selected probiotic candidates showed varied probiotic activity against *F. nucleatum*, *P. gingivalis* and *A. actinomycetemcomitans* in both the larval model an in human oral keratinocytes, warranting further investigation of the mechanisms of interaction and applicability to human health.

## Data Availability Statement

The datasets generated for this study are available on request to the corresponding author.

## Author Contributions

RM performed the laboratory-based analyses, contributed to data analysis, and co-wrote the manuscript. TC co-wrote and contributed to data analysis. CO’N, RL, and AM designed the study, supervised the project, performed the data analysis, and co-wrote the manuscript.

## Conflict of Interest

The authors declare that the research was conducted in the absence of any commercial or financial relationships that could be construed as a potential conflict of interest.
